# The role of antimicrobial prophylaxis in the management of snakebite: a systematic review

**DOI:** 10.12688/wellcomeopenres.24701.2

**Published:** 2026-05-11

**Authors:** Samuel Moody, Alison Beech, Eli Harriss, Thomas Lamb

**Affiliations:** 1 Department of Infection, North Manchester General Hospital, Manchester, England, M85RB, UK; 2 University of Oxford Health Care Libraries, Oxford, England, OX37DQ, UK; 3 Centre for Tropical Medicine and Global Health, University of Oxford Nuffield Department of Medicine, Oxford, England, OX37BN, UK

**Keywords:** Snakebite, envenoming, bacterial infection, antibiotic, antimicrobial, prophylaxis

## Abstract

**Background:**

Snakebite, a neglected tropical disease disproportionately affecting lower-income countries, is a significant cause of morbidity and mortality worldwide. Local toxicity and necrosis may result in secondary bacterial infection of bite sites. Despite most guidelines not recommending prophylactic antimicrobials, their use is common in practise. This review aims to systematically assess literature around the use of prophylactic use of antimicrobials in snakebite. We also aim to assess the incidence of secondary infections, types of antimicrobials used, and the aetiology of infections arising from snakebite.

**Methods:**

Systematic database searches for studies assessing prophylactic antimicrobial use undertaken. Data was assessed by two reviewers and extracted using a standardised proforma. Included studies were assessed for risk of bias and data extracted narratively. The protocol was prospectively registered on PROSPERO database (CRD42023430752).

**Results:**

492 studies were screened. Four met the inclusion criteria, totalling 696 patients across three countries. No study found a statistically significant benefit for the use of antibiotic prophylaxis, with no effect on the number or severity of adverse incidents. There were 114 adverse incidences related to secondary infection, with nineteen positive cultures.

**Conclusions:**

This review finds little evidence pertaining to the use of antimicrobial prophylaxis. Studies were highly heterogenous with incomplete reporting of standardised processes. Bacteria isolated supports existing observational data implicating bacteria found in snake oral flora (e.g. *M. morganii* and *Enterococcus* spp). This is an important consideration when deciding empirical antimicrobial regimens for suspected superadded infection. International consensus is required to define infection following snakebite and further high-quality research is required to draw definitive conclusions regarding antimicrobial prophylaxis.

## Introduction

Snakebite is classed as a neglected tropical disease (NTD), estimated to cause 81,000–138,000 deaths every year and leaving 400,000–2 million surviving victims. ^1^ ^–^ ^3^ Low- and middle-income countries shoulder 95% of all cases, with rural areas particularly at risk (commensurate to human interaction and encroachment onto snake habitats). ^4^ Without prompt antivenom administration patients are often left with permanent physical disfigurement: long term disabilities such as amputations, contractures and chronic ulceration are well documented. These also carry a significant socioeconomic impact given the predominance of bites in younger people. ^5^

Snake venom is a complex mixture of endo- and polypeptides, metalloproteinases, phospholipases, and endogenous autocoids (such as histamine, 5-hydroxytriptamine and kinins) and exhibit considerable inter- and intra-species variation. ^6^ ^,^ ^7^ While snakebites are known for neurotoxic or coagulopathic sequelae, bite-adjacent tissue damage is the leading cause of morbidity. Myo- and cytotoxins cause local tissue necrosis, exacerbated by tissue ischaemia from local thrombosis. ^6^ Treatment methods such as tourniquet application can worsen this local toxicity by concentrating the venom in the affected limb. ^8^

Distinguishing venom induced cytotoxicity from secondary bacterial infection in bite-adjacent tissue is challenging with many features present for both conditions: for example, pain, induration, lymphadenitis and elevated biochemical markers (such as total white cell count and C-reactive protein). Given this diagnostic challenge it is unsurprising the reported incidence of snakebite infection in literature varies widely (from 0–94%). In one systematic review and meta-analysis, aggregated prevalence of infection following snakebite was 27.0%, with high heterogeneity (I ^2^ = 99.7%). ^9^ The lack of accepted case definition further complicates the identification and comparison of snakebite infection.

The most common pathogens to cause secondary infections post-bite (isolated from wound, abscess or blood cultures) are *Morganella morganii*, *Enteroccus feacalis*, *Staphylococcus aureus*, *Proteus* spp, *Klebsiella* spp, *Aeromonas hydrophilia* and *Pseudomonas aeruginosa.* ^9^ ^,^ ^10^ Although most of these pathogens are atypical causes of skin and soft tissue infections, this closely correlates with the oral microbiota of snakes demonstrated in observational studies. ^11^ ^,^ ^12^

In recognition of the unique challenges of diagnosing snakebite associated infection, the World Health Organisation (WHO) recommends antimicrobial prophylaxis in snakebite patients in specific circumstances. ^13^ Despite some variations national snakebite guidelines are broadly in line with this WHO guidance, noting routine antimicrobial prophylaxis following snakebite is unnecessary. ^14^ ^–^ ^16^ Given the diversity in culpable snake species, challenges with microbiological culture and paucity in research, guidelines are largely reliant on expert opinion rather than evidence. ^7^ ^,^ ^17^ In practice however, antimicrobials are much more commonly prescribed as prophylaxis (and in some circumstances administered to all victims of snakebite). ^18^ ^,^ ^19^

Against the backdrop of increasing antimicrobial resistance, the indication for prophylactic antimicrobials is becoming increasingly scrutinized. Antimicrobial resistance in pathogens found in snake oral flora is common, including frequently used antimicrobials such as amoxicillin-clavulanate and 3rd generation cephalosporins. ^20^ ^,^ ^21^ Furthermore, isolates cultured from snakebite wounds and abscesses have been identified as carrying emerging resistance mechanisms- including extended spectrum beta lactamases and carbapenamases. ^22^

This systematic review aims to assess available literature around the use of antimicrobial prophylaxis in snakebite. It aims to assess the incidence of secondary infection, the rates and types of antimicrobial prophylaxis used, and the microbiological aetiology of infections arising from snakebites.

## Methods

The following bibliographic databases and trial registries were searched by an information specialist (EH) for studies, published from database inception to the search date (22.11.2023), to answer the review question: MEDLINE, Embase, and Global Health (via Ovid search platform); Scopus; Web of Science Core Collection; WHO Global Index Medicus; the Cochrane Central Register of Controlled Trials; clinicaltrials.gov; and the WHO International Clinical Trials Registry Platform. The databases were searched using relevant thesaurus terms as well as free text terms and synonyms to search the title, abstract or keyword fields, with no limits applied beyond a filter for the study type. Highly sensitive search strategies designed by Cochrane were used to search Ovid MEDLINE and Ovid Embase. ^23^ The full search strategies can be found in file ‘Extended data 1’ in the online repository. ^24^ All references were exported to Covidence systematic review tool.

### Inclusion criteria

The review’s inclusion criteria, methodology and outcomes were prospectively registered to the online systematic review platform PROSPERO. Only randomised control trials (RCT’s) in humans that compared antimicrobial prophylaxis with placebo following snakebite envenoming were included for review. There were no restrictions on the trial participants (including age, gender, ethnicity, location or health status) or date of publication. Studies were required to have pre-specified their criteria for the diagnosis of infection. Studies were excluded if their full text could not be found.

The review was designed prospectively and without prior knowledge of available literature. The authors sought to include the highest available evidence, namely RCT only, to reduce the impact of bias and confounding of the search results.

### Study selection, quality and data extraction

**Figure 1.**
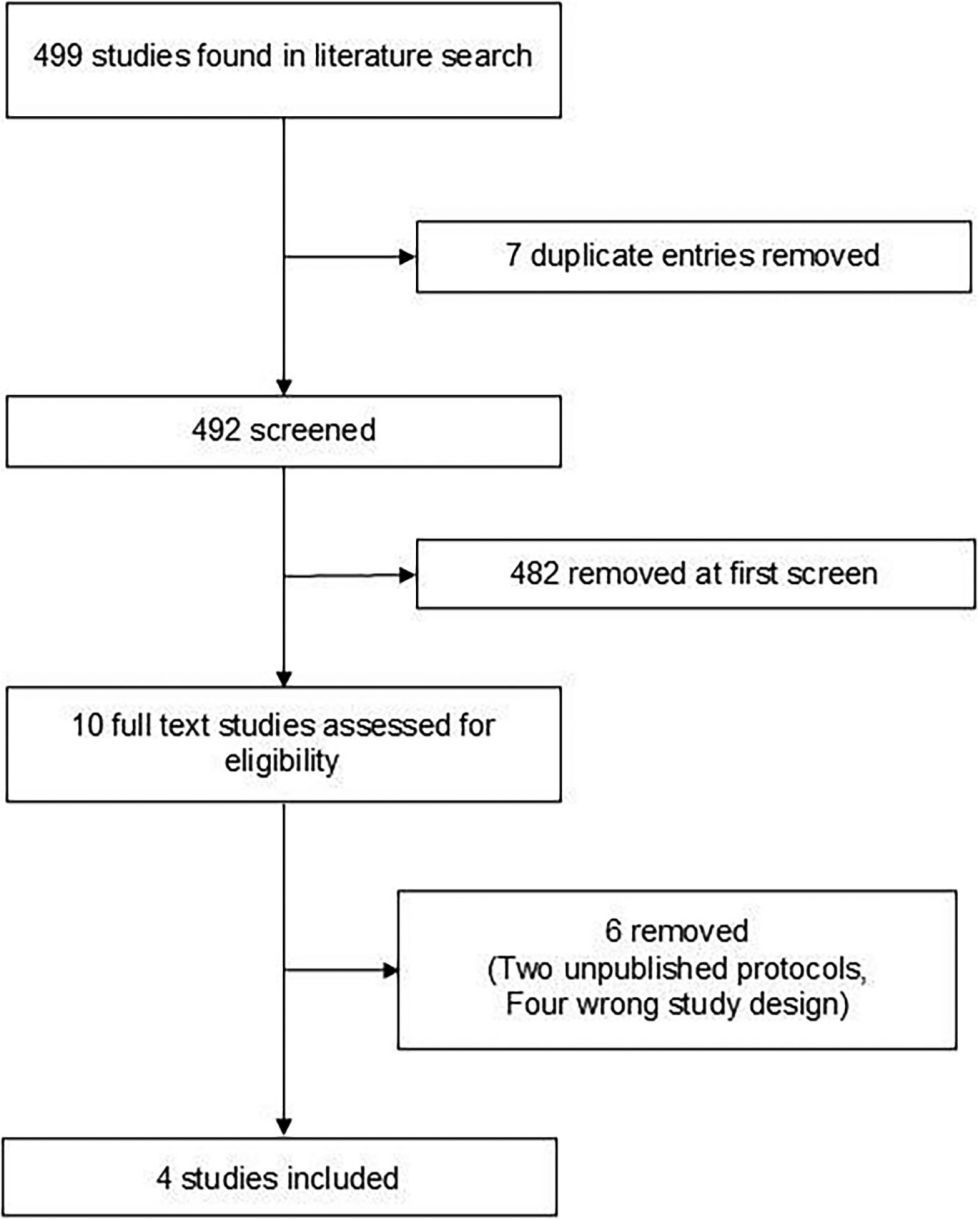
PRISMA diagram of literature search.

All studies identified were deduplicated and collated using the automated functionality of an online systematic review manager. ^25^ Each entry was reviewed independently by two reviewers (SM and AB) to determine whether it met the inclusion criteria. Discrepancies in the two reviews were arbitrated by an independent third reviewer (TL). Figure 1 details the PRISMA diagram for the search. Data was extracted using standardised, pre-designed proformas. Included papers were assessed for risk of bias using the Revised Cochrane risk-of-bias method (RoB 2). ^26^

## Results

499 articles were screened using the methodology above; of these, 7 were duplicates and were manually removed. 482 entries were unanimously removed on first screening of title and abstract; of the remaining ten, six were removed after full-text screening (two being protocols of unpublished trials and four being of the wrong study design). Four papers met the inclusion criteria. Search outcomes can be found in extended data 2. ^24^ ^,^ ^27^ ^–^ ^30^ Given the search outcome, data is presented narratively.

The four studies were conducted in three countries (Brazil, Ecuador and Sri Lanka). Two studies, ^27^ ^,^ ^28^ both in Brazil, exclusively target *Bothrops* (n = 437). Of these, 187 were species-confirmed via enzyme immunoassay, 91 were identified as *B. jararaca* (via physical confirmation) and 160 were a clinical diagnosis. One study assumed all bites to be from Pit vipers (family *Viperidae*, subfamily *Crotalinae*) based on local epidemiology. ^29^ The final study made no attempt to speciate the snakes in question but does note the four commonest snakes in their region. ^30^ The studies included a total of 696 patients: 343 receiving antimicrobial prophylaxis and 353 receiving placebo (or no treatment). The choice, route and duration of prophylactic antimicrobials varied amongst the four included studies. 114 adverse incidents (AI) relating to secondary infection (cellulitis and abscess formation) were recorded; 54 in intervention groups and 60 in control groups. None of these studies reported adverse events to the administration of antimicrobials.

Relevant findings from the included articles are compared in Table 1.

**Table 1.** Relevant findings from research included in review.

First author, year	Sachett, 2017	Jorge, 2004	Kerrigan, 1997	Kularatne, 1997
**Country**	Brazil	Brazil	Ecuador	Sri Lanka
**Trial participants**	187 (93 intervention, 94 control)	251 (122 intervention, 129 control)	114 (59 intervention, 55 control)	144 (69 intervention, 75 control)
**Definition of infection**	Abscess (presenting purulent secretion or serous-purulent secretion) or cellulitis (presence of erythema, oedema, bruising and pain, with fever, leukocytosis, lymphangitis and/or lymphadenitis) until seven days after hospitalisation	Presence of abscess (defined as the presence of pus in the region of the bite)	Clinical judgement of ‘infectious complications’ (all of which underwent surgical debridement)	Based on ‘inflammatory swelling’ (measured at sire of bite and every 5 cm, to 25 cm proximally, and compared to matched controls)
**Antimicrobial intervention**	Oral amoxicillin clavulanate 875/125 mg, twice daily, seven days	Oral chloramphenicol 500 mg, four times a day, five days	IV gentamicin 1 mg/kg, three times daily AND IV chloramphenicol 12 mg/kg, four times a day, 24 hours	IV benzylpenicillin 2 megaunits, four times a day AND IV metronidazole 500 mg, three times a day, five days
**Snake species and number identified (if reported)**	*Bothrops* spp.	*Bothrops* spp.	*Crotalinae*	105 ‘viper’, 39 ‘others’
**Method of snake identification**	Enzyme immunoassay	91 via physical specimen, 160 on toxidrome	43 identified as *Bothrops* spp (method not documented). Remaining cases not specified.	Not documented
**Adverse incidents related to infection**	64 Cellulitis and 29 abscess complications in total 74 patients. 35.5% in intervention group, 44.1% in control group (95% CI: 0.56–1.15, P = 0.235)	12 abscesses: 6 intervention (4.9%), 6 control (4.7%), P ≥ 0.05	9 abscesses: 6 intervention (10.2%), 3 control (5.5%), P = 0.558	No difference in inflammatory swelling. Improvement in swelling a D5 observed in 91.3% in intervention group and 93.3% in control group (P = 0.65)
**Microbiologically confirmed infections**	6	2	11	Not assessed
**Bacteria isolated & breakdown by trial group**	*M.morganii* (5), *S.aureus* (1). Trial group breakdown not documented.	Intervention group: *E.coli* (1). Control group: *M.morganii* (1)	Intervention group: *E.coli* (3), *S.aureus* (3), *Klebsiella spp* (1). Control group: *Klebsiella spp* (1), *Enterobacter spp* (1), *Proteus spp* (1), *S.aureus* (1)	NA
**Outcome: benefit of prophylactic antimicrobials**	No benefit	No benefit	No benefit	No benefit

AI: Adverse incidents; NA: not applicable.

None of these studies found a statistically significant benefit in the use of prophylactic antimicrobials following a snakebite injury, with their use having no effect on the number or severity of AI’s related to infection. There was considerable difference in the incidence of secondary infection across the studies, ranging from 7.9 to 39.6% (one study’s reporting methods made calculating infection incidence impossible). ^30^ Each study used their own predefined definition of secondary infection.

### Microbiological aetiology

Nineteen positive bacterial cultures (eight from intervention groups, five from control groups and six not disclosed) were reported from all 695 participants. One study did not assess microbiological aetiology, ^30^ and another used aerobic culture only. ^29^ *M. morganii* was the most frequently identified bacterial pathogen (n = 7), followed by *E. coli* (n = 4) and *S. aureus* (n = 4). Two papers reported bacterial susceptibility data. ^27^ ^,^ ^29^

### Test of bias (RoB-2)

Two were found to have low risk and two were found to have ‘some concerns’ of bias ( Table 2): One provided no information on their allocation sequence or document a pre-specified analysis plan for their findings (domains 1 & 5), ^30^ and the other did not note their data analysis plan (domain 5). ^29^ Two studies performed prospective power calculations to estimate sample size, ^27^ ^,^ ^30^ of which one study recruited sufficient participants according to this calculation. ^27^

**Table 2.** Summary of Risk-Of-Bias (ROB-2) analysis of articles included in review. ^25^

Study	1. Randomisation process	2. Deviations from intended intervention	3. Missing outcome data	4. Measurement of the outcome	5. Bias in the selection of the reported results	Overall risk of bias
Jorge	Low risk	Low risk	Low risk	Low risk	Low risk	Low risk
Sachett	Low risk	Low risk	Low risk	Low risk	Low risk	Low risk
Kerrigan	Low risk	Low risk	Low risk	Low risk	Some concerns ^1^	Some concerns (domain 5)
Kularantne	Some concerns ^2^	Low risk	Low risk	Low risk	Some concerns ^1^	Some concerns (domains 1&5)

^1^ No information recorded regarding randomisation or concealment of allocation sequence.

^2^ No information regarding a pre-specified analysis plan for final data (which should be finalised before unblinding).

## Discussion

This systematic analysis finds little peer-reviewed evidence concerning the efficacy or utility of routine administration of antimicrobial prophylaxis in snakebite envenoming. All included articles conclude there is no benefit in prophylaxis preventing secondary bacterial infections. This conclusion is further supported by a recent observational study of rattlesnake ( *Crotalus* spp) envenoming in Arizona, USA which identified snakebite associated infection in 20 of 2059 (0.97%) incidences of envenoming. ^31^ This is an important finding given the recommendations for prophylaxis by multiple international guidelines.

Two of the included articles have concerns over risk of bias and there is considerable heterogeneity (including the clinical setting, responsible snake species and definition of snakebite) making them unsuitable for meta-analysis. There are many potential reasons for this: key factors such as local cytotoxicity, wound necrosis, oral flora, local susceptibility data and antibiotic choice may impact on the efficacy of antibiotic prophylaxis it is not possible to confidently delineate which factors contribute to the heterogeneity. Given the small number of studies meeting the inclusion criteria, we did not believe that subgroup analyses would add value to the manuscript.

Antimicrobial choice is important and must ensure adequate broad-spectrum coverage; we note little consensus on the drugs used here. The antimicrobials used in these four studies (chloramphenicol, co-amoxiclav, combination gentamicin/chloramphenicol and combination benzylpenicillin/metronidazole) were likely selected on the basis of their relative broad-spectrum gram positive, gram negative and anaerobic activity; however, Sachett *et al.* notes the poor efficacy of co-amoxiclav in preventing secondary infections. ^27^ Although Kerrigan *et al.* notes all gram-negative rods and gram-positive cocci isolated are sensitive to gentamicin, ^29^ some guidelines advocate against the use of aminoglycosides due to risk of nephrotoxicity or potential exacerbation of the effects of neurotoxic envenoming. ^16^ Coupled with wider concerns around antimicrobial administration- cost, side-effect risk, and importantly risk of antimicrobial resistance- their routine use becomes harder to justify. It is unfortunate the none of these studies reported adverse events to the administration of antimicrobials, or insights into antimicrobial resistance, which are pertinent information when weighing up the risks and benefits of their prophylactic use.

The limited reporting of wound sampling and laboratory procedures makes interpretation difficult and highlights the need for further dedicated research in the area. In one study cultures were only taken from patients with an abscess and only presenting to one study site (which enrolled just 12 of the 251 patients in the study). ^28^ The other studies do not report their procedures for taking cultures (or how many were taken), making it impossible to calculate accurate positivity rates. An absence of anaerobic culture capability is also likely to undermine reporting incidence ^29^ – which is particularly pertinent given the polymicrobial nature of animal-bite associated infections. ^32^ Future studies should include a clear clinical case definition, document robust sampling methodology and operational procedures for both aerobic and anaerobic cultures.

The bacterial isolates reported in these studies were in keeping with other reports of snakebite associated infection. ^7^ The lack of anaerobic bacteria identified in the included studies may reflect methodological errors of the included studies (and hinders conclusions on anaerobic isolates). Although isolates cultured from abscesses and wounds include bacteria from animal oral flora (e.g. *Morganella morganii*), it is uncertain their relative significance in infection pathogenesis. ^33^

There are limitations to the evidence found by this review. Our searches exclude ‘grey literature’ sources not amenable to our search methodology (for example, the WHO’s Global Index Medicus). While every effort has been made to ensure methodological purity, we accept that this entails risk of narrowing search findings (or distancing our findings from clinical practice, which RCT’s may not be representative of ). The exclusion of other research modalities, such as observational trials, was deemed necessary to reduce potential bias or confounding in our findings. Searches were also only made for research in the English language, potentially further restricting our evidence base and risking language bias.

The heterogeneity of included studies also suggests a wider lack of term definitions, possibly obfuscating systematic search findings. We are also unable to draw broader conclusions about snakebite from the studies reported here. Only one subfamily of vipers – *Crotalinae* – have been reported, with no other species positively identified. The included data therefore excludes snake genera such as *Naja* spp. that is more commonly associated with snakebite associated infection. Furthermore, snake oral flora is unique, containing bacterial organisms not commonly observed in routine soft tissue infections (coupled with a mechanism for deep inoculation and concomitant tissue inflammation); more research is required around both species and geography. Consensus in case definition for snakebite associated infection, and comprehensive yet pragmatic laboratory procedures, would increase the external validity of future studies. Details of polymicrobial infections and antimicrobial susceptibility patterns to aid empirical antimicrobial decision making is increasingly important and should be considered in future research design. Our observations cannot be extended to other geographical areas, necessitating the need for replicable studies performed in multiple locations. This highlights the importance of standardisation of definitions and laboratory processes.

While this review was being prepared for publication, a further non-inferiority trial was published comparing routine vs. clinically directed use of co-amoxiclav in snakebite in southern India. ^34^ The study randomized 66 patients, with those in the clinically directed arm (n = 34) being assessed daily and receiving antimicrobials if they were found to have any of several pre-determined clinical or biochemical complications. It speciated 26 (39.4%) bites; 21 as Russell’s viper and 5 as saw-scaled vipers. Importantly, these are both from a subfamily of vipers ( *Viperinae*) not described in the studies included in this review. Like the four included studies, it had a bespoke definition of secondary infection (based on surgical intervention, abscess formation or worsening limb swelling), and noted no adverse events related to the use of antimicrobials.

This trial was stopped prematurely due to the Covid-19 pandemic; non-inferiority of clinically directed antibiotics use was not demonstrated, but the study was only able to recruit around 50% of the planned sample size. Total antimicrobial consumption was significantly lower in the directed arm vs the prophylactic arm. There was one death recorded in each group. This study has not been integrated into the review to conserve methodological purity, but is evidence that the topic of prophylactic antimicrobial use remains a relevant topic for research worldwide (this is the first research, to our knowledge, undertaken in India). It also adds to the body of research concluding there is no evidence in the use of prophylactic antimicrobials.

## Conclusion

We find a paucity of quality research concerning the role of antimicrobial prophylaxis in snakebite. Of the evidence found, there is no evidence for the routine administration of antimicrobial prophylaxis following snakebite at reducing bacterial superinfection. Coupled with the global efforts to reduce antimicrobial administration (and its effects on antimicrobial sensitivity), this adds to the reasoning against the use of prophylactic antimicrobial use in snakebite. Larger and geographically diverse trials with standardised infection definitions are required to build up a more inclusive picture of secondary infection rates, and if antimicrobial prophylaxis is effective in reducing this number. While such research is challenging given the wide heterogeneity of both snake species and their oral microbiota, ongoing and recently published research is a signal that this topic remains worthy of both investment and investigation.

## Transparency declaration

The authors affirm that this manuscript is an honest, accurate, and transparent account of the study being reported; that no important aspects of the study have been omitted; and that any discrepancies from the study as planned (and, if relevant, registered) have been explained.

## Data availability statement

Zenodo: Extended data: The role of antimicrobial prophylaxis in the treatment of snakebite: a systematic review, https://doi.org/10.5281/zenodo.17171619. ^24^

This project contains the following underlying data:

• Extended_data_1 (search strategies)
• Extended_data_2 (Search outcome data)
• PRISMA_2020_Main_Checklist (PRISMA checklist)
• PRISMA_2020_Abstract_Checklist (PRISMA abstract checklist).

Data are available under the terms of the Creative Commons Attribution 4.0 International license (CC-BY 4.0).
