# Supplementary material for: The role of antimicrobial prophylaxis in the management of snakebite: a systematic review
Source: Wellcome Open Res. 2026 May 11;10:554. Originally published 2025 Oct 6. [Version 2] doi: 10.12688/wellcomeopenres.24701.2 (PMC12640490; doi:10.12688/wellcomeopenres.24701.2)
Supplement: Peer review history

# Reviewer report: Reviewer response for version 2

**DOI:** 10.21956/wellcomeopenres.29135.r155234

**Published:** 2026-05-19

**Authors:** Nicholas Amani Hamman

**Affiliations:** 1 Community Medicne and Public Health, Gombe State University, Gombe, Nigeria

I thank the authors for the considerable effort invested in revising this manuscript. The revised version is noticeably improved in clarity, methodological transparency, data presentation and interpretation of findings.

 The authors have addressed the major concerns I raised in the previous review(1).

 The manuscript now provides clearer details regarding the search strategy and study selection process, improved presentation of infection definitions and outcome data. It also offers a more balanced discussion of heterogeneity, geographical limitations and the overall strength of the evidence. The discussion and conclusion sections are now more appropriately cautious and better aligned with the available data.

 I also appreciate the inclusion of additional discussion around the recently published Indian trial as well as the improved explanation of the limitations associated with the small number and heterogeneity of included studies.

 However, I believe a few minor editorial issues may still benefit from attention prior to final indexing . A final proofread for minor grammatical inconsistencies and overly long sentences in parts of the Introduction and Discussion would further improve readability. The authors should also ensure consistent formatting throughout the manuscript especially regarding italicization of bacterial species names (very important and relevant please) and uniform naming/style of antimicrobial agents across the text and tables. In addition, a final check of citation and reference formatting for journal style consistency would be helpful.  However these are minor issues and do not affect the scientific merit of the manuscript.

 Overall, this manuscript addresses an important and clinically relevant question in snakebite management, particularly in low- and middle-income settings where antimicrobial use remains common despite limited evidence.

 In my opinion, the manuscript now represents a useful and balanced synthesis of the currently available randomized evidence on prophylactic antimicrobial use following snakebite envenomation.

 I believe the manuscript is suitable for indexing in its current form.

Are the rationale for, and objectives of, the Systematic Review clearly stated?

Yes

Is the statistical analysis and its interpretation appropriate?

Partly

If this is a Living Systematic Review, is the ‘living’ method appropriate and is the search schedule clearly defined and justified? (‘Living Systematic Review’ or a variation of this term should be included in the title.)

Not applicable

Are sufficient details of the methods and analysis provided to allow replication by others?

Yes

Are the conclusions drawn adequately supported by the results presented in the review?

Partly

Reviewer Expertise:

Epidemiology and control of Snakebite Envenoming; Maternal and Child Health

I confirm that I have read this submission and believe that I have an appropriate level of expertise to confirm that it is of an acceptable scientific standard.

# Reviewer report: Reviewer response for version 1

**DOI:** 10.21956/wellcomeopenres.27217.r137363

**Published:** 2025-11-22

**Authors:** Jaideep C. Menon

**Affiliations:** 1 Amrita Institute of Medical Sciences, Kochi, Kerala, India

I read with interest the manuscript authored by Moody and colleagues titled 'The role of antimicrobial prophylaxis in the management of snakebite: a systematic review', and have the following onservations to make

 The topic is relevant as National Guidelines vary substantially on advocacy towards the use of anti-microbials and drug.

 Given the pro-inflammatory nature of certain snake venom components, it becomes particulalry difficult to diagnose underlying infection, given that whole blood counts, neutrophilia and raised acute phase reactants are asscoaited with snakebite envenoming.

 Additionally significant local bite site infalmmation maked it difficult to diagnose superadded infection other than from a culture.

 All these together make it difficult to diagnose superadded infection with snakebite envenoming. The Systematic Review though not powered by great numbers does shed some light on this issue.

 I would like the authors to touch on a few of these points for grater clarity in the context of SBE.

 I opine that the manuscript is well written and finds my approval for indexing.

Are the rationale for, and objectives of, the Systematic Review clearly stated?

Yes

Is the statistical analysis and its interpretation appropriate?

Yes

If this is a Living Systematic Review, is the ‘living’ method appropriate and is the search schedule clearly defined and justified? (‘Living Systematic Review’ or a variation of this term should be included in the title.)

No

Are sufficient details of the methods and analysis provided to allow replication by others?

Yes

Are the conclusions drawn adequately supported by the results presented in the review?

Yes

Reviewer Expertise:

Clinical aspects and epidemiology of snakebite envenoming in India

I confirm that I have read this submission and believe that I have an appropriate level of expertise to confirm that it is of an acceptable scientific standard.

# Reviewer report: Reviewer response for version 1

**DOI:** 10.21956/wellcomeopenres.27217.r137361

**Published:** 2025-11-10

**Authors:** Joseph J Erinjery

**Affiliations:** 1 Kannur University, Mananthavady Campus, Mananthavady, Kerala, India

Snakebite is considered a neglected tropical disease, and it affects millions of lives and livelihoods around the world. The current systematic review assesses the efficacy of prophylactic antimicrobials for the treatment of secondary infections at bite sites. As the authors noted, only a few existing studies were available for the review, and some of those selected were biased. Based on these studies, the authors do not recommend using antimicrobials at bite sites. The article is well written, and I only have a few minor comments. 

 - The study could have used some meta-analysis methods for analysis

 - Italics for species names in the Microbiological aetiology section.

Are the rationale for, and objectives of, the Systematic Review clearly stated?

Yes

Is the statistical analysis and its interpretation appropriate?

Partly

If this is a Living Systematic Review, is the ‘living’ method appropriate and is the search schedule clearly defined and justified? (‘Living Systematic Review’ or a variation of this term should be included in the title.)

Not applicable

Are sufficient details of the methods and analysis provided to allow replication by others?

Yes

Are the conclusions drawn adequately supported by the results presented in the review?

Yes

Reviewer Expertise:

Disease prevalence; snake bite prevalence;

I confirm that I have read this submission and believe that I have an appropriate level of expertise to confirm that it is of an acceptable scientific standard.

# Reviewer report: Reviewer response for version 1

**DOI:** 10.21956/wellcomeopenres.27217.r135568

**Published:** 2025-10-17

**Authors:** Nicholas Amani Hamman

**Affiliations:** 1 Community Medicne and Public Health, Gombe State University, Gombe, Nigeria

**REVIEW REPORT**

**Manuscript title: The role of antimicrobial prophylaxis in the management of snakebite: a systematic review**

**General summary**

 This manuscript presents a systematic review assessing the efficacy of prophylactic antimicrobial use following snakebite envenomation. The authors registered their protocol on PROSPERO, searched multiple databases and identified four randomized controlled trials (RCTs) totaling 696 participants. Their main finding is that prophylactic antibiotic administration does not significantly reduce the incidence or severity of secondary bacterial infection after snakebite.

 The topic is highly relevant especially for low- and middle-income countries where snakebite burden and antimicrobial misuse are common. However, despite the relevance, the manuscript has methodological, structural and interpretative weaknesses that limit its current scientific robustness. While the authors have followed several PRISMA principles(1), key details necessary for reproducibility, critical appraisal and transparency are either underreported, briefly summarized or insufficiently detailed

  Major Comments

**Search strategy transparency and comprehensiveness**

 The manuscript describes an extensive database search but crucial methodological details are missing:

1. The full search strategy is said to be available as “Extended Data 1” yet no summary or example query is included in the main text.
2. There is no date for the last search, which violates PRISMA 2020(1) reporting standards.
3. No mention of grey literature sources, regional databases (e.g., LILACS, African Index Medicus) or manual reference checks which are essential given the geographical burden of snakebite (South Asia, sub-Saharan Africa).
4. Limiting inclusion to English-language studies introduces a potential language bias especially as snakebite trials are often published in Portuguese, Spanish, Hindi or Thai.

**Recommendation:**

 Include the complete search syntax in an appendix or supplement. Specify the exact final search date. The authors acknowledge the English only limitation in the discussion but they should also explain its rationale and possible implication for the comprehensiveness of the review

**Inclusion/exclusion criteria**

 Restricting inclusion to RCTs is excessively stringent for this field where RCTs are rare due to ethical and logistical barriers. This restriction results in only four included studies which undermines the generalizability of findings and may distort the real-world clinical picture.

 While methodological purity is admirable, excluding high-quality observational or quasi-experimental studies leads to a narrow and potentially unrepresentative evidence based that may not reflect clinical practice

**Recommendation**:

 Authors should justify this decision explicitly in the Methods or preferably consider a sensitive analysis or expanded literature review including a well design cohort or prospective studies to increase the breath and relevance of evidence

**Data extraction and risk-of-bias evaluation**

 The manuscript claims to use standardized proformas and independent reviewers but:

1. There is no mention of inter-rater reliability (e.g., kappa coefficient or percentage agreement).
2. The RoB 2 summary table lacks supporting narrative justifications as the text merely reproduces domain ratings without explaining the reasoning behind each risk judgment.
3. There is no discussion of publication bias assessment (e.g., funnel plot, Egger’s test) even if meta-analysis was not feasible, narrative bias assessment is expected.

**Recommendation:**

 Include detailed rationale for each RoB 2 domain judgment in supplementary material. If a quantitative assessment was not possible add a short narrative discussion of potential publication bias

**Handling of heterogeneity and justification for narrative synthesis**

 The authors state that “data are presented narratively” due to high heterogeneity, but this is not quantified. The decision not to attempt a quantitative synthesis should be justified more explicitly using at least descriptive commentary or visual comparison across studies.

 Moreover:

1. There is no clear definition of what dimensions (population, intervention, outcome definitions or snake species) contributed to heterogeneity.
2. No attempt was made at subgroup analysis (e.g., by snake genus or antibiotic type), even though limited exploratory comparisons could have been informative

**Recommendation:**

 Provide explicit justification for narrative synthesis and summarize the main sources of heterogeneity in tabular or narrative form. If possible, include exploratory subgroup analyses or at least tabular comparisons of infection incidence and relative risk.

**Incomplete data presentation**

 Several crucial data points are omitted:

1. Infection definitions vary between studies but these definitions are not summarized in the Results.
2. There is no unified table summarizing infection incidence (in %), relative risks or confidence intervals for each included trial.
3. No information on adverse events from antibiotic use (e.g., gastrointestinal symptoms, allergic reactions), which is relevant when weighing prophylactic risk-benefit balance.

**Recommendation:**

 Reformat Table 1 or create an additional summary table including:

1. Definition of infection used per study.
2. Infection incidence per arm.
3. Relative risk or risk difference.
4. Adverse event rates.

This will improve interpretability and transparency.

**Discussion**

 While the discussion is generally cautious, there are several issues:

1. The authors extrapolate findings from four small, geographically limited trials (Brazil, Ecuador, Sri Lanka) to all snakebite contexts globally, which is overly broad given ecological and microbiological diversity.
2. The mention of “recent observational data” and “new trials in India” is helpful but not systematically integrated i.e. the Indian trial (ref 34) should be more critically compared to the included studies rather than discussed in isolation
3. The discussion on antimicrobial resistance (AMR) is conceptually important but not well linked to the actual evidence extracted from the included trials (none of which investigated resistance profiles comprehensively).

**Recommendation:**

 Tighten the discussion by explicitly separating evidence derived from included studies vs. contextual or theoretical considerations. Avoid generalizing findings beyond the included snake species or settings.

**Limitations section**

 While the limitations are mentioned, several important points are not sufficiently expanded:

1. Exclusion of grey literature and non-English publications.
2. Lack of standard case definition for infection across trials.
3. Absence of anaerobic cultures and incomplete microbiological reporting.
4. Small sample size and low event rate reducing statistical power.

**Recommendation:**

 Expand the limitations to reflect these methodological constraints and explicitly note that these factors reduce the certainty and generalizability of the conclusions

**Conclusions and implications**

 The conclusion that “routine antimicrobial prophylaxis should not be recommended” is too definitive given the evidence base. The absence of evidence for benefit is not evidence of absence of benefit, especially with only four small, heterogeneous RCTs.

**Recommendation:**

 Rephrase to reflect uncertainty:

 “Current randomized evidence does not demonstrate a significant benefit from routine antimicrobial prophylaxis after snakebite; however, the available studies are small and heterogeneous. Larger, geographically diverse trials using standardized infection definitions are required before definitive clinical recommendations can be made.”

**Minor Comments**

**Writing and structure**

1. Some sentences in the introduction are overly long and densely referenced. Consider tightening prose for readability.
2. Ensure all abbreviations (e.g., AI, RoB, PROSPERO) are defined at first use.

**Tables and figures**

1. Table 1 should include column totals for clarity.
2. . include the date of the search.

**Referencing**

1. Verify reference numbering consistency; some citations (e.g., 24, 27–30) appear misplaced or duplicated.
2. The recent Indian non-inferiority trial (ref 34) should include full citation details and journal reference if available

**Formatting**

1. Ensure all bacterial species names are italicized (e.g., Morganella morganii).
2. Use consistent formatting for antimicrobial agents (generic names only, lowercase)

**Supplementary material**

1. Extended data references should include working links or DOI numbers to ensure reproducibility.

** Conclusion**

 Major revision

 While the manuscript addresses an important and underexplored clinical question, clarifications and expansions in methodology, data presentation and conclusion phrasing are needed before it can be considered for publication. Specifically:

1. Provide full search transparency and clarify inclusion rationale.
2. Expand data presentation with infection incidence and risk estimates.
3. Refine conclusions to match evidence strength.

Are the rationale for, and objectives of, the Systematic Review clearly stated?

Yes

Is the statistical analysis and its interpretation appropriate?

Partly

If this is a Living Systematic Review, is the ‘living’ method appropriate and is the search schedule clearly defined and justified? (‘Living Systematic Review’ or a variation of this term should be included in the title.)

Not applicable

Are sufficient details of the methods and analysis provided to allow replication by others?

Yes

Are the conclusions drawn adequately supported by the results presented in the review?

Partly

Reviewer Expertise:

Epidemiology and control of Snakebite Envenoming; Maternal and Child Health

I confirm that I have read this submission and believe that I have an appropriate level of expertise to confirm that it is of an acceptable scientific standard, however I have significant reservations, as outlined above.

## References

[1] : The PRISMA 2020 statement: An updated guideline for reporting systematic reviews. *Journal of Clinical Epidemiology* .2021;134: 10.1016/j.jclinepi.2021.03.001 178-189 10.1016/j.jclinepi.2021.03.001 33789819
